# Oral contraceptive pill use and the susceptibility to markers of exercise-induced muscle damage

**DOI:** 10.1007/s00421-017-3629-6

**Published:** 2017-05-11

**Authors:** K. M. Hicks, G. Onambélé-Pearson, K. Winwood, C. I. Morse

**Affiliations:** 1 Department of Sport, Exercise and Rehabilitation, Northumbria University, Newcastle City Campus, 2 Ellison Place, Newcastle upon Tyne, NE1 8ST UK; 2 Health Exercise and Active Living Research Centre, Exercise and Sport Science, Manchester Metropolitan University, Crewe, CW1 5DU UK

**Keywords:** Exercise-induced muscle damage, Oral contraceptive pill, Tendon properties

## Abstract

**Purpose:**

Firstly, to establish whether oral contraceptive pill (OCP) users are more susceptible to muscle damage compared to non-users, and secondly, to establish whether differences can be attributed to differences in patella tendon properties.

**Methods:**

Nine female OCP users and 9 female non-users participated in the investigation. Combining dynamometry, electromyography and ultrasonography, patella tendon properties and vastus lateralis architectural properties were measured pre and during the first of 6 sets of 12 maximal voluntary eccentric knee extensions. Serum oestrogen levels were measured on the 7th day of the pill cycle and the 14th day of menstrual cycle in OCP users and non-users, respectively. Maximal voluntary isometric knee extension torque loss, creatine kinase and muscle soreness were measured 48 h pre-damage, post-damage, and 48, 96 and 168 h post-damage.

**Results:**

Oestrogen levels were significantly lower in OCP users compared to non-users (209 ± 115 and 433 ± 147 pg/ml, respectively, *p* = 0.004). Proposed determinants of muscle damage, patella tendon stiffness and maximal eccentric torque did not differ between OCP users and non-users. The change in creatine kinase from pre to peak was significantly higher in OCP users compared to non-users (962 ± 968 and 386 ± 474 Ul, respectively, *p* = 0.016). There were no other differences in markers of muscle damage.

**Conclusion:**

Although our findings suggest that, when compared to non-users, the OCP may augment the creatine kinase response following eccentric exercise, it does not increase the susceptibility to any other markers of muscle damage.

## Introduction

Exercise-induced muscle damage (EIMD) has been shown to be lower in females compared to males (Joyce et al. 2014; Sewright et al. 2008). It has been suggested that this attenuated EIMD is due to the direct antioxidant properties of oestrogen (Carter et al. 2001). However, an indirect role of oestrogen in suppressing EIMD, through altering the tendon properties in females, has recently been proposed (Hicks et al. 2016).

The role of oestrogen in attenuating EIMD has been assessed through the manipulation of circulating levels in animals and has been shown to attenuate EIMD in both male and female rats (Amelink et al. 1990, 1991; Bär et al. 1988). For example, Bär et al. (1988) reported that the increase in creatine kinase (CK) post-EIMD, in both males and ovariectomised female rats, was suppressed by oestrogen supplements prior to EIMD. Alternatively, within human studies, oestrogen levels can be manipulated through the use of synthetic hormones [e.g. hormone replacement therapy and the oral contraceptive pill (OCP)].

The OCP down regulates oestrogen levels throughout the menstrual cycle by altering the hypothalamic–pituitary–ovarian feedback loop, thus inhibiting the peak in oestrogen at ovulation (Elliott-Sale et al. 2013; Van Heusden and Fauser 2002). Consequently, women who take the OCP have significantly lower levels of circulating oestrogen compared to non-users throughout the menstrual cycle (Bryant et al. 2008; Fleischman et al. 2010). Due to the protective properties of oestrogen and the suppressed oestrogen levels in OCP users, it is suggested that OCP users are more susceptible to EIMD; however, the literature remains inconclusive with evidence supporting (Carter et al. 2001; Joyce et al. 2014; Minahan et al. 2015) or refuting (Sewright et al. 2008; Thompson et al. 1997) an increased susceptibility to EIMD. For example, in support of an increased susceptibility to EIMD, 48-h post-maximal eccentric leg extensions Joyce et al. (2014), reported significantly higher levels of serum CK levels in males and female OCP users compared to female non-users. Whereas, following maximal eccentric contractions of the elbow flexors, Sewright et al. (2008) reported no significant difference in CK between OCP users and non-users. The differences in exercise protocols/exercising muscle groups (Chen et al. 2011) and different phases of the menstrual cycle/oestrogen levels, make it difficult to determine the true role of the OCP on EIMD.

In addition to acting as an antioxidant, oestrogen may indirectly attenuate EIMD through altering tendon properties. Compliant tendons have been evidenced to mitigate fascicle lengthening (Hicks et al. 2013) and attenuating peak forces and torques during eccentric contractions (Hoffman et al. 2014; Roberts and Azizi 2010; Roberts and Konow 2013). Therefore, it is suggested that lower tendon stiffness may act as a mechanical buffer and attenuate levels of EIMD (Hoffman et al. 2014; Roberts and Azizi 2010; Roberts and Konow 2013).

High oestrogen levels have been reported to decrease tendon stiffness by attenuating the rate of tendon collagen synthesis (Hansen et al. 2009a, b). In parallel, IGF-I has an anabolic effect on tendon fibroblasts by increasing collagen syntheses in a dose-dependent manner (Olesen et al. 2006) and hence would be assumed to be associated with higher tendon stiffness. Interestingly, oestrogen is associated with decreased IGF-I bioavailability (Heald et al. 2005). In other words, a lower tendon stiffness would be expected in the presence of elevated oestrogen due to the sequestering away of IGF. Within OCP users, however, despite lower oestrogen levels compared to non-users, it remains unclear, with arguments supporting (Bryant et al. 2008) or contesting (Hansen et al. 2013) differences in tendon properties between OCP users and non-users. It is possible, however, that the aforementioned discrepancies between Bryant et al. (2008) and Hansen et al. (2013) findings may be attributed to differences in the number of available oestrogen receptors expressed on the Achilles and patella tendon.

Therefore, the aim of the current study was twofold: firstly, to establish whether OCP users are more susceptible to EIMD compared to non-users and secondly, to establish whether any differences in EIMD could be attributed, in part, to differences in tendon properties.

## Materials and methods

### Participants

A total of 18 females were divided into two groups: women taking a monophasic OCP (*n* = 9, 23.4 ± 2.4 years of age, 70.0 ± 9.9 kg and 1.70 ± 0.05 m) and eumenorrheic women who had never taken the OCP [(non-users) *n* = 9, 21.2 ± 1.5 years of age, 63.2 ± 5.6 kg and 1.65 ± 0.09 m]. All participants self-reported as being recreationally active (undertaking no more than 1 h of “moderate” physical activity per week) and did not take part in any structured resistance training. All procedures complied with the Declaration of Helsinki World Medical Association (2013) and ethical approval was obtained through the local ethics committee at Manchester Metropolitan University. All women reported regular menstrual cycles, documenting an average cycle length of 28 ± 1 days. On average the OCP users had been taking a combined monophasic OCP, with an ethinyl estradiol dosage between 20 and 30 µg for 3 ± 1 years. The types of OCP used within the current study are taken daily followed by a 7-day pill-free interval. Exclusion criteria included any resistance training undertaken in the last 6 months, occupation or lifestyle that required regular heavy lifting or carrying, any known muscle disorder, the use of dietary supplements (for example, multivitamins), and any musculoskeletal injury in the last 3 months. Further exclusion criteria included, previous use of any other forms of hormone-based contraception, continuous pill OCP or mini-OCP users, irregular menstrual cycles [where regular cycles were defined as 24–36 days (Cole et al. 2009; Landgren et al. 1980)] in the last 12 months, and pregnancy in the year preceding inclusion in the current study. All inclusion and exclusion criteria were determined through participant questionnaire prior to inclusion within this investigation.

### Testing protocol

**Fig. 1:**
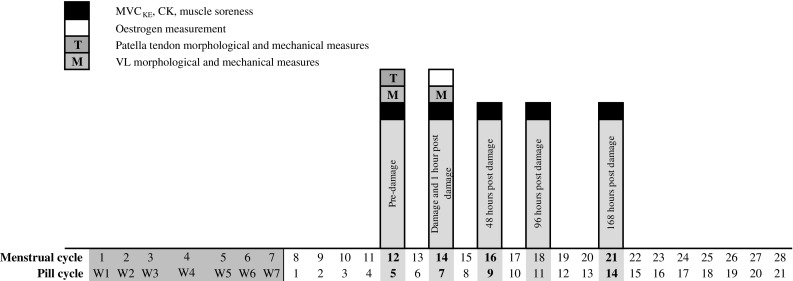
Schematic diagram to illustrate the testing timeline throughout the cycle (pill or menstrual) in OCP users and non-users. *MVC*_*KE*_ maximal voluntary isometric knee extension, *CK* creatine kinase, *VL* vastus lateralis

Once selected, participants were asked to visit the laboratory on five different occasions over 9 days. Non-users were tested on the 14th day (self-reported) of the menstrual cycle to measure oestrogen levels at ovulation (Brown 1955; Cole et al. 2009). To reflect the timing of ovulation in the non-users and to try and avoid the secondary peak in oestrogen levels (Legro et al. 2008), OCP user’s oestrogen levels were tested on the 7th pill taking day (Elliott et al. 2005). The 7th pill taking day is equivalent to the 14th day of the overall pill cycle (7 pill-free days plus 7 pill taking days), therefore the measurement day will be referred to as the 14th day of the cycle from this point forward. To avoid an acute peak in oestrogen levels the OCP users were not tested within 2 h of taking their OCP (Sneader 2005). The design and several of the measurement techniques within the current study have been reported previously (Hicks et al. 2013). The testing sessions were as follows: (1) pre-damage; (2) damage (48 h post pre-damage); (3) 48 h; (4) 96 h and (5) 168 h post-damage (see Fig. 1). Pre-damage assessments consisted of stature and mass (anthropometric measures), patella tendon moment arm, 5–6 ml blood sample (for serum CK levels), dynamometer familiarisation (within pre-damage), morphological and mechanical measures of the patella tendon (tendon size and stiffness, respectively) and maximal voluntary isometric knee extension (MVC_KE_) torque measurements at six angles [60°, 65°, 70°, 75°, 80° and 90° (full extension = 0°)]. Participants performed two practice maximal voluntary contractions, at two knee joint angles during the familiarisation session. Stature and mass were measured using a wall-mounted stadiometer (Harpenden, Holtain Crymych, UK) and digital scales (Seca model 873, Seca, Germany), respectively. The damage session was on the 14th day of the cycle (pill or menstrual) in OCP users and non-users. The damage session consisted of maximal voluntary eccentric knee contractions (MVE_KE_), 5–6 ml venous blood sample (for serum CK and oestrogen levels), rating of muscle soreness and MVC_KE_ torque measurements. 48, 96 and 168 h testing session consisted of 5–6 ml blood sample (for serum CK levels), rating of muscle soreness, and MVC_KE_ torque measurements at six angles (60°, 65°, 70°, 75°, 80° and 90°).

All tests were carried out in the non-dominant leg. The non-dominant leg was defined as the leg that provided stability during movements such as kicking a ball. Participants were seated in an isokinetic dynamometer (Cybex Norm, Cybex International, NY, USA), with a hip angle of 85°. To reduce any extraneous movement during maximal efforts, participants were secured in a seated position using inextensible straps secured around the shoulders and hips. The isokinetic dynamometer axis of rotation was visually aligned with the knee joint’s centre of rotation. During pre-damage, the isokinetic dynamometer settings (chair position, distance from the dynamometer, dynamometer arm length) and anatomical zero (leg fully extended, knee angle 0°) were recorded to ensure accurate repeatability in the following sessions.

### Vastus lateralis anatomical cross-sectional area

The method used to determine vastus lateralis (VL) cross-sectional area has been reported in detail previously (Hicks et al. 2013). In brief, in a supine position, VL anatomical cross-sectional area (VL_ACSA_) was measured using a real-time B-mode ultrasound (AU5 Harmonic, Esaote Biomedica, Genoa, Italy). At 50% of VL muscle length echo-absorptive markers (Transpore surgical tape, Medisave UK Ltd, UK), were placed in parallel at intervals of 30 mm. The ultrasound probe (7.5 MHz linear array probe, 38 mm wide), was held perpendicular to the VL muscle in the axial plane, and moved steadily over the echo-absorptive markers from the lateral to the medial edge of the muscle. The images were recorded in real-time at 25 frames per second (Adobe Premier pro Version 6, Adobe Systems Software, Ireland). Using video editing software (Adobe Premier Elements, version 10), still images were acquired at each 30 mm interval. The shadows cast by the echo-absorptive markers allowed the neighbouring still images to be aligned, thus reconstructing the entire VL_ACSA_ in a single image (Adobe Photoshop Elements, version 10). Digitising software (ImageJ 1.45, National Institutes of Health, USA) was used to measure VL_ACSA_.

### Torque measurements

At six different knee angles [60°, 65°, 70°, 75°, 80° and 90° (full extension = 0°)], participants were instructed to perform two MVC_KE_ lasting ~2 s at the plateau, with 90 s rest between contractions. Torque was presented, in real-time, on a Macintosh G4 computer (Apple Inc., Cupertino, CA, USA), via an A/D converter (Biopac Systems, Santa Barbara, CA, USA). Torque measurements were later analysed offline with the accompanying software (Acknowledge, version 3.9.2). The highest peak torque produced at each angle was taken as MVC_KE_ peak torque. The knee angle where the highest torque was produced was recorded as optimal knee angle. In a randomised order, MVC_KE_ torque measurements were repeated at all six knee angles 60 min post-eccentric exercise [to reduce any fatigue effect (Walsh et al. 2004)] and 48, 96 and 168 h post-eccentric exercise. MVC_KE_ torque loss was calculated from optimal knee angle identified at pre-damage.

### Patella tendon length and cross-sectional area

A real-time B-mode ultrasound (AU5 Harmonic, Esaote Biomedica, Genoa, Italy) was used to measure patella tendon cross-sectional area and patella tendon length at a fixed 90° knee angle. The distance between the apex of the patella and the tibial tuberosity, marked using sagittal ultrasound images, was taken as patella tendon length. To measure patella tendon cross-sectional area, the ultrasound probe was placed in the transverse plane and images were captured at 25, 50, and 75% of patella tendon length. The images were later analysed offline using image analyses software, ImageJ (1.45, National Institutes of Health, USA) patella tendon cross-sectional area is presented as a mean of all three images (O’Brien et al. 2010).

### Patella tendon stiffness

The participants were seated in the isokinetic dynamometer, with the knee angle fixed at 90°, and were instructed to perform a ramped, isometric MVC_KE_ lasting ~5–6 s. Ramped MVC_KE_ torque and displacement of the patella tendon were synchronised using a 10-V square wave, signal generator. Patella tendon displacement was measured over two MVC_KE_, once with the ultrasound probe positioned over the distal edge of the patella and on the second contraction over the tibial tuberosity (Onambélé et al. 2007), so that total displacement would be computed from the composite of proximal and distal patella motions (see below). Torque was presented on a Macintosh G4 computer (Apple Inc., Cupertino, CA, USA), via an A/D converter and subsequently analysed with the accompanying software (Acknowledge, Biopac Systems, Santa Barbara, CA, USA). To create an external marker on the ultrasound images, an echo-absorptive marker was placed on the skin. Using the marker to calculate displacement, the distance of the marker (shadow) from an anatomical reference point at the beginning of the contraction to the position of the shadow at the end of the contraction was calculated. A square wave, signal generator was used to synchronise the ultrasound images with the torque acquisition system. Images were captured at ~10% intervals of ramped MVC_KE_ torque (Onambélé et al. 2007). Total patella tendon displacement was calculated as displacement at the apex of the patella plus the displacement at the tibial tuberosity (Onambélé et al. 2007). Patella tendon forces were calculated as: (MVC_KE_ torque + antagonist co-activation torque)/patella tendon moment arm.

Patella tendon moment arm was measured at 90° (full extension = 0°) in the sagittal plane, from a dual-energy X-ray absorptiometry scan (frame 23.3 cm × 13.7 cm, Hologic Discovery, Vertec Scientific Ltd, UK), and subsequently analysed using a DICOM image assessment tool (OsiriX DICOM viewer, ver. 4.0, Pixemo, Switzerland). Patella tendon moment arm length was determined as the perpendicular distance from the centre of the patella tendon to the tibio–femoral contact point. Dual-energy X-ray absorptiometry scans have recently been shown to be a reliable and valid method of measuring patella tendon moment arm length when compared to the gold standard method (MRI) (Erskine et al. 2014).

For the calculation of tendon force, the calculation of antagonist co-activation torque is described below. The patella tendon force–elongation curve constructed from data analysed at every 10% MVC_KE_, was then fitted with a second-order polynomial function forced through zero (Onambélé et al. 2007). The tangential slope at discreet sections of the curve, relative to MVC_KE_ force, was calculated by differentiating the curve at every 10% patella tendon force interval. In addition, to standardise the comparison of patella tendon stiffness at an absolute load, the slope of the tangential line, corresponding to the MVC_KE_ force of the weakest participant, was computed for each subject.

### Co-activation during maximal voluntary isometric knee extension

In order to compute patella tendon force, the co-activation of the biceps femoris during MVC_KE_ was measured. To determine co-activation of the biceps femoris during the ramped MVC_KE_, electrodes (Ambu, Neuroline 720, Denmark) were placed in a bipolar configuration at 25% of biceps femoris muscle length (distal end = 0%). A reference electrode (Ambu, Blue Sensor, Denmark) was placed on the lateral tibial condyle. The raw electromyography signal was amplified (×2000) and filtered (through low and high pass filters of 10 and 500 Hz, respectively, notch at 50 Hz) with the sampling frequency set at 2000 Hz. Ramped MVC_KE_ torque and biceps femoris electromyography were recorded in real-time and synchronised using a (10-V) square wave signal generator. Participants performed two maximal voluntary isometric knee flexions (MVC_KF_) at 90°. They were instructed to perform MVC_KF_ rapidly and as forcefully as possible and instructed to relax once a 2-s plateau had been attained (as observed on the dynamometer screen display). The root mean square of the biceps femoris electromyography signal was calculated 500 ms either side of instantaneous MVC_KF_ maximal torque from the contraction corresponding to the highest MVC_KF_ torque. Prior to contraction the baseline signal noise was calculated as the integral root mean square over 1 s and removed from the measured electromyography during MVC_KF_ and MVC_KE_. At every 10% of ramped MVC_KE_ torque the absolute integral of the biceps femoris electromyography was taken over 250 ms. Co-activation torque was calculated as: (biceps femoris electromyography during ramped MVC_KE_/biceps femoris electromyography during MVC_KF_) × MVC_KF_ torque at 90° knee angle (Onambélé et al. 2007). This equation assumes that the biceps femoris is representative of the entire hamstring (Carolan and Cafarelli 1992) and that a linear relationship exists between biceps femoris electromyography and MVC_KF_ torque (Lippold 1952).

### Patella tendon stress/strain relationship

Patella tendon strain was calculated as a ratio of total patella tendon displacement and patella tendon length. Patella tendon stress was calculated as: patella tendon force (N)/patella tendon cross-sectional area (mm^2^).

### Young’s modulus

Young’s modulus was calculated as: patella tendon stiffness × [patella tendon length (mm)/patella tendon cross-sectional area (mm^2^)].

### ‘Damaging’ eccentric exercise

The damaging protocol used within the study has been described in detail previously (Hicks et al. 2013). Following a warm-up of 10 isokinetic knee extensions and knee flexions, participants performed six sets of 12 MVE_KE_. For the eccentric exercise, the knee extension range of motion was set at 20°–90° (0° = full extension). The eccentric phase of the contractions was performed at an isokinetic angular velocity of 30°/s (Jamurtas et al. 2005). The concentric phase was performed passively at an angular velocity of 60°/s. Two minutes rest was provided between each set. Participants remained seated in the isokinetic dynamometer throughout the entire exercise protocol, including rest periods. Visual feedback and verbal encouragement were continuously provided throughout the protocol. MVE_KE_ torque was recorded throughout each contraction and displayed via the torque acquisition system. For each set, peak MVE_KE_ torque was determined as the highest torque out of the 12 repetitions. Average peak MVE_KE_ torque was calculated as an average of peak MVE_KE_ across six sets.

### Muscle soreness

Muscle soreness was measured using a visual analogue scale. The visual analogue scale consisted of a 100 mm line, with 0 mm labelled “No pain at all” and 100 mm labelled “Unbearable pain”. Seated in the isokinetic dynamometer, the leg was passively moved through a full range of motion at 30°/s. Participants were asked to mark a line perpendicular to the visual analogue scale to denote the level of pain they experienced during the passive movement. The visual analogue scale has been reported to be a reliable measure of muscle soreness [ICC > 0.96 (Bijur et al. 2001)].

### Blood samples

Venous blood samples were taken to measure CK and oestradiol levels. A 21-gauge needle was inserted into the antecubital vein of the forearm using a 10 ml syringe. 5–6 ml of blood was drawn into a serum collection tube. The sample was left on a crushed ice bed for 60 min. The sample was then centrifuged at 4500 rpm at 0 °C for 10 min. Using a 200–1000 µl pipette (Eppendorf, Hamburg, Germany), the resulting serum sample was separated into three aliquots (~500 µl each) and stored in eppendorfs at −20 °C until CK and oestradiol analysis was performed.

Creatine kinase levels were measured using a standard colorimetry procedure, measuring at optical density 340 nm (BioTek ELx800 96 well Microplate Reader) and immediately analysed (Gen5, version 2.0). Each sample was run in duplicate-quadruplets using an EnzyChrom^TM^ CK Assay Kit (BioAssay Systems, Hayward, CA, USA, sensitivity 5 U/l, manufacturer intra-assay variability ≤5%). An average of 2–4 readings were taken. Creatine kinase activity is reported in two ways, firstly as absolute values and secondly, as the increase from pre to peak CK over the 168 h [(ΔCK_peak_), i.e. the peak CK value − the pre-CK values].

Oestradiol was measured using a standard enzyme-linked immunosorbent assay (ELISA) procedure (Alpha Diagnostic International, San Antonio, USA). Absorbance was measured at optical density of 450 nm (BioTek ELx800 96 well Microplate Reader). The minimal oestradiol detection was ~10 pg/ml, the manufacturers intra-precision and inter-precision was 9.85 and 10.3%, respectively. To calculate oestrogen levels, a standard curve was plotted using the six standards against their absorbance. Using the mean absorbance of each sample, the concentration of the sample was read directly from the standard curve.

### Statistics

Statistical analyses were carried out using the statistical software package SPSS (v.19, Chicago, IL, USA) for Windows and Microsoft Excel. To ensure that the data were parametric, the Levene’s and Shapiro–Wilk’s tests were used to assess the variance and normality of the data. If parametric tests were violated, the equivalent non-parametric tests were used. For group differences in anthropometric measures, oestrogen levels, VL_ACSA_, patella tendon properties, and ΔCK_peak_ independent *T* tests were used. A 2 × 5 mixed design analysis of variance [ANOVA, between factors: OCP use (2 levels) and time from EIMD (5 levels)] was used for muscle soreness, MVC_KE_ torque loss and CK levels. Wherever the assumption of sphericity was violated, the Greenhouse–Geisser correction was used. When a significant group effect was found a planned contrast, with LSD correction, was used to identify where the significant difference lay. Since an association was reported between age and MVC_KE_ torque loss, an analysis of covariance was used. For the variables being investigated as a potential determinant of EIMD, confidence levels and Cohen *d* were calculated. Significance was set at *p* ≤ 0.05. The data are presented as mean ± standard deviation.

## Results

### Anthropometric measurements

OCP users were significantly older by 2.2 years (*p* = 0.010) and of an 11% greater mass (*p* = 0.047) than non-users. There was no significant difference in stature between OCP users and non-users (*p* = 0.061).

### Oestrogen levels

Serum oestrogen levels were significantly lower in OCP users compared to non-users on the 14th day of the cycle (pill or menstrual) (209 ± 115 and 433 ± 147 pg/ml, respectively, *p* = 0.004).

### VL_ACSA_

There was no significant difference in VL_ACSA_ between OCP users and non-users (21.9 ± 2.64 and 20.1 ± 3.40 cm^2^, respectively, *p* = 0.224).

### Tendon properties

**Fig. 2:**
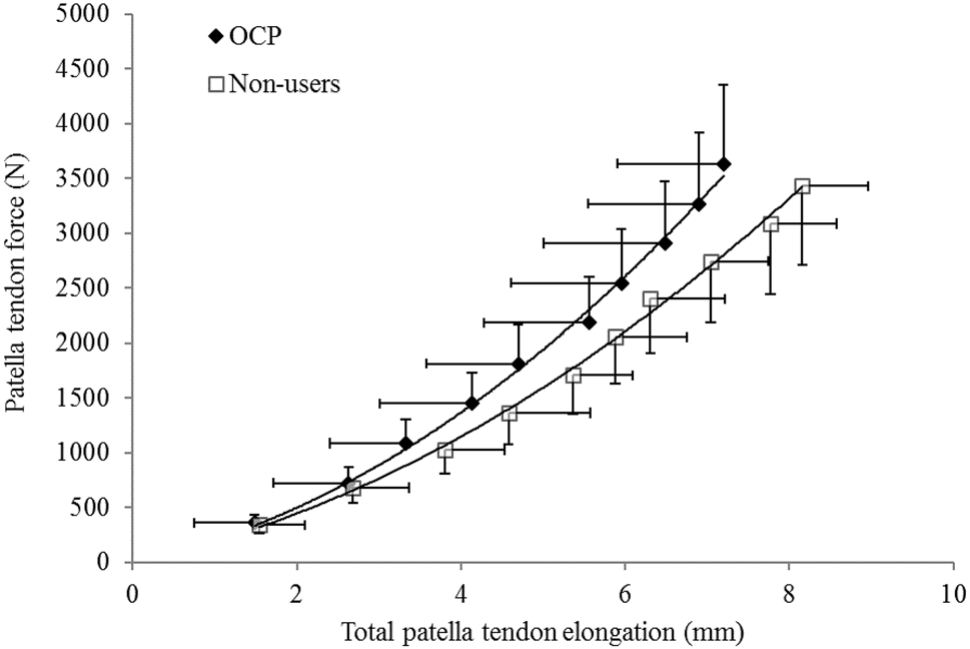
Patella tendon force–elongation relationship, during ramped maximal voluntary isometric contraction, in oral contraceptive pill user and non-users. Data are presented as mean ± standard deviation

Patella tendon properties for OCP users and non-users are presented in Table 1. Patella tendon length, patella tendon cross-sectional area and patella tendon moment arm were not significantly different between OCP users and non-users (*p* ≥ 0.05). Furthermore, ramped MVC_KE_ torque and patella tendon force were not significantly different between OCP users and non-users (*p* ≥ 0.05). Patella tendon elongation was not significantly different between OCP users and non-users (7.20 ± 1.30, 8.16 ± 0.80 mm, respectively, *p* = 0.075). Tendon stiffness (Fig. 2) at MVC_KE_ was not significantly different between OCP users and non-users [*p* = 0.153, CI (−502, 88.0), *d* = 0.73]. Young’s modulus was not significantly different between OCP users and non-users [*p* = 0.213, CI (−190, 428), *d* = 0.38]. Patella tendon stiffness calculated at a standardised patella tendon force (2330 N) was not significantly different between OCP users and non-users (725 ± 219 and 565 ± 103 N/mm, respectively [*p* = 0.070, CI (−10.9, 331), *d* = 0.94]. Patella tendon Young’s modulus calculated at a standardised patella tendon force (2330 N) was not significantly different between OCP users and non-users [628 ± 219 and 536 ± 190 MPa, respectively (*p* = 0.175 CI (−111, 298), *d* = 0.453)].

**Table 1:** Patella tendon properties in OCP users and non-users

	OCP users	Non-users
Patella tendon length (mm)	52.9 ± 4.3	49.0 ± 6.0
Mean patella tendon cross-sectional area (mm^2^)	63.2 ± 15.1	56.0 ± 17.2
Patella tendon moment arm (cm)	4.01 ± 0.24	3.96 ± 0.28
Maximal ramped MVC_KE_ torque (Nm)	143 ± 30	132 ± 27
Maximal patella tendon force (N)	3634 ± 717	3430 ± 768
Maximal patella tendon stiffness (N/mm)	872 ± 366	665 ± 169
Maximal Young’s modulus (MPa)	761 ± 331	642 ± 285

Patella tendon stiffness and Young’s modulus are presented at 100% ramped maximal voluntary isometric knee extension. Patella tendon moment arm was measured at 90° knee angle

### Torque production during MVE_KE_

There was no significant difference in the average peak MVE_KE_ torque of each set (six in total) between OCP users and non-users (*p* ≤ 0.05). There was no significant difference in the average peak MVE_KE_ torque (averaged over 6 sets) between OCP users and non-users (182 ± 33 and 167 ± 32 Nm, respectively, *p* = 0.093). Peak MVE_KE_ torque made relative to pre-MVC_KE_ torque was significantly higher in OCP users compared to non-users (108 ± 16.7 and 85.0 ± 11.9%, respectively, *p* = 0.002).

### Change in optimal knee angle

Pre-damage, optimal MVC_KE_ knee angle was not significantly different between OCP users and non-users (median, 75.0 ± 7.0°, and 75.0 ± 7.0°, respectively, *p* = 0.430). Post-EIMD both OCP users (80.0 ± 7.0°, *p* = 0.010) and non-users (median 80.0 ± 7.0°, *p* = 0.030) demonstrated a significant rightward shift in MVC_KE_ optimal angle. There was no significant difference in the magnitude of the rightward shift in MVC_KE_ optimal angle between OCP users and non-users (*p* = 0.311).

### MVC_KE_ torque loss

**Fig. 3:**
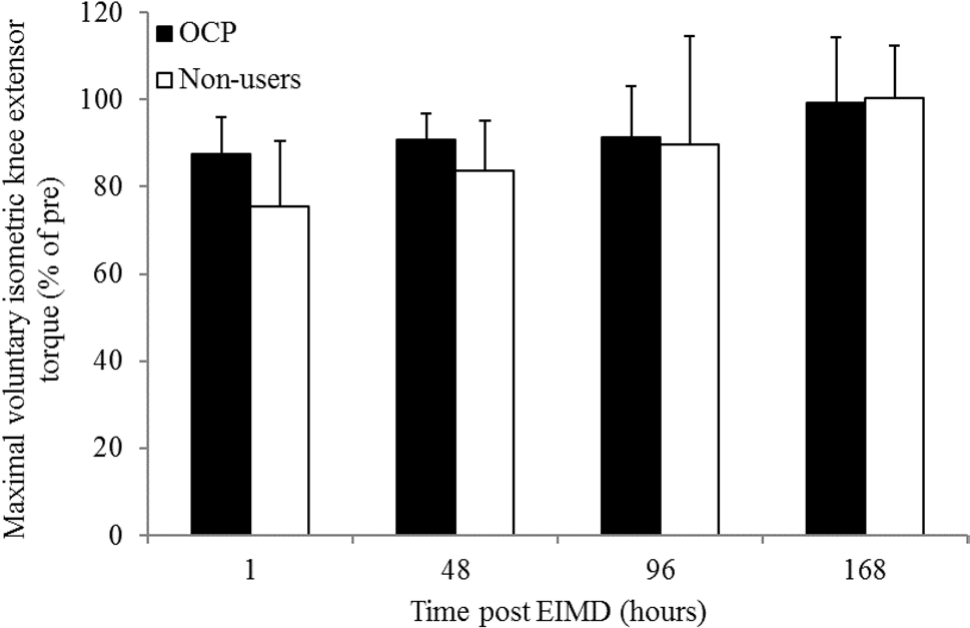
Maximal voluntary isometric knee extensor torque in oral contraceptive pill users and non-users expressed as a percentage of maximal voluntary isometric knee extensor torque pre-damage. Data are presented as mean ± standard deviation

MVC_KE_ torque loss expressed as a percentage of pre-damage MVC_KE_ torque is illustrated in Fig. 3. A two-way repeated mixed ANOVA for MVC_KE_ torque loss post-EIMD, reported a significant main effect of time (*p* = 0.0004); however, no group effect (OCP users versus non-users *p* = 0.257) nor interaction (times versus group, *p* = 0.118) was reported. Pre-MVC_KE_ peak torque was not significantly different between OCP users and non-users (169 ± 24.3 and 194 ± 36.2 Nm, *p* = 0.055). Although a significant tendency was reported, peak MVC_KE_ torque loss made relative to pre-damage MVC_KE_ torque was not significantly different between OCP users compared to non-users (17 ± 7 and 28 ± 13%, respectively [*p* = 0.055, CI (−21.5, 10.5), *d* = 0.94)].

A significant correlation was observed between age and MVC_KE_ torque loss (*r* = 0.58, *p* = 0.011). Age was therefore considered as a covariate of MVC_KE_ torque loss. A subsequent ANCOVA revealed that no significant difference in MVC_KE_ torque loss between OCP users and non-users remained when age was accounted for (*p* = 0.179).

### Creatine kinase levels

**Fig. 4:**
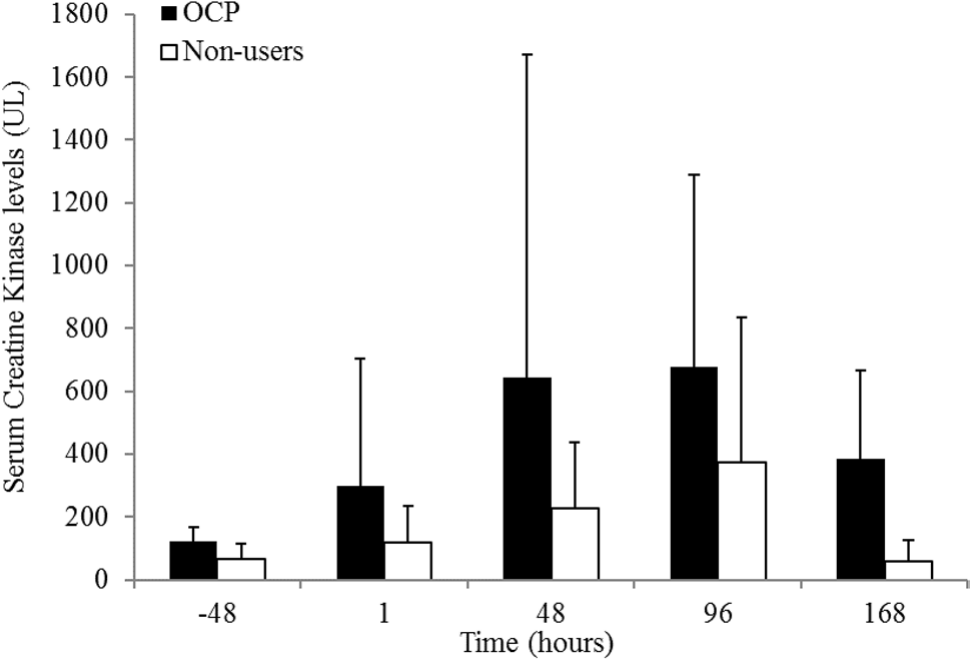
Creatine kinase response in oral contraceptive pill users and non-users pre (−48 h) and post-exercise induced muscle damage. Data are presented as mean ± standard deviation

A two-way repeated mixed ANOVA for CK reported a significant main effect of time (*p* = 0.040); however, no significant group (OCP users versus non-users, *p* = 0.057) or interaction (time versus group, *p* = 0.576) effects were reported (Fig. 4). ΔCK_peak_ was significantly higher in OCP users compared to non-users over the 168 h [962 ± 968 and 386 ± 474 UL, respectively (*p* = 0.016, CI (−186, 1338), *d* = 0.76)].

### Muscle soreness

A two-way mixed measures ANOVA for muscle soreness reported a significant main effect of time (*p* = 0.0004); however, there was no significant group difference (OCP users versus non-users, *p* = 0.598) or an interaction effect (*p* = 0.127). For both OCP users and non-users peak muscle soreness occurred 48 h post-EIMD. There was no significant difference in peak muscle soreness between OCP users and non-users (40 ± 3 mm and 41 ± 1 mm, respectively, *p* = 0.358).

## Discussion

The current study found that (1) the CK response was augmented in OCP users compared to non-users; however, MVC_KE_ torque loss and muscle soreness were not different; (2) the patella tendon properties were not significantly different between the OCP users and non-users and, as such, did not contribute to the observed significant difference in EIMD.

Within the current study, the circulating oestrogen levels within the OCP users were 2.8 times higher than oestrogen levels within OCP users reported by Bryant et al. (2008). This discrepancy in oestrogen levels may be confounded by a secondary rise in oestrogen levels following the pill-free interval period within the current study. Despite elevated levels within OCP users, in agreement with previous research (Bryant et al. 2008), the current study confirms a significant down regulation of oestrogen within OCP users compared to non-users. Thus it is reasonable to assume that in the present study, the down regulation of oestrogen in the OCP users compared to the non-users and the suppression of oestrogen in the OCP users, remained throughout the cycle (Bryant et al. 2008).

Due to high levels of oestrogen attenuating circulating levels of IGF-I (Heald et al. 2005) it can be expected that the anabolic effect of IGF-I on tendon fibroblast and therefore tendon collagen synthesis is greater in OCP users compared to non-users. Subsequently, higher tendon stiffness in OCP users compared to non-user may be expected. Within the current study, however, despite significantly lower oestrogen levels in OCP users, there was no significant difference in patella tendon stiffness in vivo between OCP users and non-users. Although our data show a trend towards higher stiffness in the OCP users, our findings did not reach significance and contradict those of Bryant et al. (2008), but are in agreement with those of Hansen et al. (2013). Bryant et al. (2008) concluded that exposure to the OCP (≥1 year) decreases Achilles tendon strain properties. These discrepancies may be attributed to the fact that Bryant et al. (2008) reported data from the Achilles tendon, whereas the present study assessed the patella tendon. Therefore, it is possible that the Achilles and patella tendons show inconsistent responses to the OCP due to the difference in the number of oestrogen receptor sites, as our findings concur with those of Hansen et al. (2013). Hansen et al. (2013) reported no significant difference in patella tendon stiffness or Young’s modulus between OCP users and non-users. The current study confirms that the OCP does not alter patella tendon stiffness or Young’s modulus in recreationally active participants. The conclusion that the OCP has no significant effect on tendon properties, specifically patella tendon stiffness, may discount any potential differences in EIMD between OCP users and non-users being directly attributed to patella tendon properties.

As a marker of muscle damage, the current study reported no significant difference in MVC_KE_ torque loss between OCP users and non-users, which both supports (Sewright et al. 2008) and contradicts (Minahan et al. 2015; Savage and Clarkson 2002) previous research. Within the knee extensors, our findings directly contradict those of Minahan et al. (2015) who reported torque loss 48 h post-EIMD to be significantly higher in OCP users compared to non-users. The discrepancies may be attributed to Minahan et al. (2015) reporting substantially lower levels of oestrogen in the OCP users compared to the current study. Differing levels of oestrogen may be explained by Minahan et al. (2015) measuring oestrogen during the 7 days pill-free period, whereas the current study measured oestrogen on the 14th day (7th pill day) and the mix of OCP brands used within both studies (Elliott-Sale et al. 2013). Although the current study tested on the 7th day of the pill cycle to try and avoid the secondary peak in oestrogen, testing on the 10th day of the pill cycle (Legro et al. 2008) and controlling the type of OCP used (Elliott-Sale et al. 2013) may be required to ensure OCP oestrogen levels are plateaued and comparable, respectively. Therefore, although a significant difference in oestrogen levels between OCP users and non-users was reported within the current study, the augmented oestrogen levels within the OCP users when measured on day 7 of the pill cycle may have masked the effect of OCP use on torque loss post-EIMD. Therefore, the current study concludes that on the 14th day of the cycle (pill or menstrual), there is no significant difference in MVC_KE_ torque loss between OCP users and non-users post-EIMD.

Although torque loss is regarded as the most functional measure of EIMD (Warren et al. 1999), in agreement with previous research (Joyce et al. 2014; Minahan et al. 2015; Roth et al. 2001), the current study reported ΔCK_peak_ to be significantly higher in OCP users (low oestrogen levels) compared to non-users (high oestrogen levels) following EIMD. Although Joyce et al. (2014) and Minahan et al. (2015) reported CK levels to be significantly higher in OCP users compared to non-users, they only monitored CK up to 48 h post-EIMD despite peak CK characteristically occurring at 96 h post-EIMD (Hyatt and Clarkson 1998). By measuring CK at 96 and 168 h post-EIMD, the current study reduces the chance of missing a true peak in CK and can therefore confirm that ΔCK_peak_ is significantly higher in OCP users compared to non-users post-EIMD. In apparent contrast to our findings, 72 h post-EIMD, Carter et al. (2001) observed OCP users to have significantly lower CK levels compared to non-users. It should be noted, however, that we carried out EIMD on day 14 of the menstrual cycle, whereas Carter et al. (2001) carried out EIMD on day 22 (midluteal phase). In the present study, we observed oestrogen levels to be significantly lower in OCP users compared to non-users, whereas Carter et al. (2001) showed OCP users to higher oestrogen levels compared to non-users. Ignoring the OCP status of Carter et al. (2001) participants, at the time of testing, the group with the lowest oestrogen level showed significantly higher CK levels post-EIMD. This is therefore consistent with our findings, that the lower oestrogen levels (observed within our OCP users) results in a higher ΔCK_peak_ post-EIMD.

Oestrogen has been reported to decrease cell membrane fluidity, thus increase cell membrane stability. An increase in cell membrane stability may prevent muscle proteins (i.e. CK) leaking into the interstitial space and into the systemic circulation (Carter et al. 2001; Wiseman et al. 1993) and attenuate the influx of neutrophils and cytokines post-EIMD (MacNeil et al. 2011; Silvestri et al. 2003). In support of oestrogen preventing the leakage of CK from the intracellular membrane, Carter et al. (2001) reported a moderate correlation (*r* = −0.43, *p* < 0.05) between total resting oestrogen levels and overall CK. Therefore, the potential effects of oestrogen on cell membrane stability may explain the differences in CK levels between OCP users and non-users within the current study; however, muscle biopsies would be required to confirm such conclusions.

Although the current study controlled for the type (monophasic) and the dosage of synthetic oestrogen level within the OCP, it must be noted that the oestrogen levels vary between different brands of OCP (Elliott et al. 2005). Therefore, to fully understand the effect of OCP on EIMD, a specific brand needs to be investigated. Furthermore, due to the natural fluctuation of oestrogen levels during the menstrual cycle and the repeated measure design of the current study, measurement of oestrogen levels at every time point, rather than just on day 14 of the menstrual cycle, may be required in future studies.

As no other significant differences between suggested determinants of EIMD in OCP users and non-users (tendon properties and MVE_KE_ torque) have been reported, the current study concludes that the higher ΔCK_peak_ in OCP users may be attributed to the significantly higher oestrogen levels in non-users. Therefore, the current study concludes that OCP use does not increase susceptibility to functional impairment (MVC_KE_ torque loss) post-EIMD, but it does augment the CK response post-EIMD.

## Abbreviations

ANOVA: Analysis of variance
CK: Creatine kinase
EIMD: Exercise-induced muscle damage
MVE_KE_: Maximal voluntary eccentric knee contractions
MVC_KE_: Maximal voluntary isometric knee extension
MVC_KF_: Maximal voluntary isometric knee flexions
OCP: Oral contraceptive pill
ΔCK_peak_: The increase from pre to peak CK over 168 h
VL: Vastus lateralis
VL_ACSA_: Vastus lateralis anatomical cross-sectional area
